# Correspondence between Dr. T. B. Gunning and the Census Bureau

**Published:** 1881-01

**Authors:** 


### ARTICLE II.

### *Correspondence between Dr. T. B. Gunning and the Census Bureau.*

The following correspondence will show to what degree the dental profession is indebted to Dr. Thos. B. Gunning, of New York City, for relief from an unwarranted demand for statistics, on the ground that dental offices are "establishments of productive industry," and dental practitioners required to furnish statistics of manufactures to the Census Bureau. Dr. Gunning's clear and able letter convinced the Superintendent of the Census Bureau that such a demand was made through pure ignorance of what dentistry is, and the result is that the profession generally has been saved from a great annoyance and trouble, by the Superintendent of the Census, on account of the informa tion furnished by Dr. Gunning, having determined to omit from the tabulations of his office dentistry in any of its branches.

DEPARTMENT OF THE INTERIOR.—CENSUS OFFICE.

*Office of Chief Special Agent,*

21 Courtland Street,

New York, Dec. 14th, 1880.

Dr. Thos. B. Gunning,

*Dentist,*

34 East 21st, St., City.

*Sir.*—You are reported as declining to give the information necessary to fill the blank handed you by my assistant, the form not being as you deem the best. I have to say that this form has been prepared by the Census Department, and we are required to obtain the information necessary to fill it from you, which the law compels you to give.

I trust therefore you will give us no further trouble in the matter, but fill the enclosed blank and resturn it to this office.

I am, yours respectfully, &c.,

Charles E. Hill,

*Chief Special Agent for the City of New York.*

---

New York, Dec. 15th, 1880.

Charles E. Hill, Esq.,

*Chief Special Agent for City of New York.*

*Sir.*—Your letter to me of the 14th tells that your assistant handed me a blank of which the form has been prepared by the Census Department. I am thus corrected in my mistake, into which I was led by the peculiarity of the blank above mentioned and by the subsequent conduct of your assistant, in supposing that he was an agent of a company claiming some patent.

When he first called I thought that he was from the Census Office, and I acceded to his request to look into the blank. Finding nothing in the paper as printed which referred to my profession—Dental Surgery, I studied the penned interlineations in red ink. My conclusions were that properly I could not say anything, and I waited for his return. When he came I suggested that perhaps I could make a statement on a schedule such as is left with surgeons and physicians; he replied, in substance, that they did not have to make any return; that there was no other schedule, and he gave me to understand that surgical operations, etc., as I might except.

After further remarks and some apparent annoyance at my endeavors to gain moreinformation, he suddenly left.

This in consideration of the fact that I had reported to the Census Agent some months before, and that although over forty years in practice, I had never heard of any special return being asked for from Dentists—led me to conclude that the blank schedule was presented to me for an illegal purpose.

In answer to the remainder of your letter I would say first, that I am very desirous to comply with every legal demand, and also save you and all connected with the Census Office from trouble so far as I know how. In your letter you say that you are required to get from me the information necessary to fill up the blank; while you also trust that I will fill it up. But you do not tell me what information you require; and in my judgment I have nothing to report upon the schedule which you have mailed to me, and I am willing to endorse this statement upon it, if you think it will facilitate matters. Awaiting your instructions, I am, sir, your obt. servt.

T. B. Gunning.

DEPARTMENT OF THE INTERIOR.—CENSUS OFFICE.

*Office of Chief Special Agent,*
21 Courtland Street,
New York, Dec. 17th 1880.

*My Dear Sir.*—I have great pleasure in owning receipt of your very full and courteous reply to my letter of the 14th. inst.

The object of the inquiries submitted to you is to ascertain the amount of industry expended upon the "mechanical" branch of Dentistry—and the form of schedule has been approved by the Census Office.

To these inquiries, a statement of the fact that one performs *no* mechanical but only surgical dentistry is, it appears to me, a sufficient answer, but you will appreciate the inability of this office, correctly to determine such a question except by actual inquiry—and even then not without some embarrassment, from the difficulty of making the matter clear to all minds. I should esteem it a favor if you would drop in here (Room 57) and grant me a personal interview, if you should be in the vicinity at any time soon, in order that my official intercourse with the members of your profession, may be in strict conformity with such suggestions as the more prominent and intelligent may offer, towards the accomplishment of the end sought. I am, sir

Very Truly Yours,
Charles E. Hill,
*Chief Special Agent.*

Thos. B. Gunning, M. D.

34 East 21st. St.,

New York City.

---

New York, Dec. 24th. 1880.

Charles E. Hill, Esq.,
*Chief Special Agent, Census Office.*

*Sir.*—I have the pleasure to acknowledge the receipt of your answer to my letter of the 16th inst.

I am unable to call at your office in time to make the suggestions available which you have so politely invited me to offer, and especially as they refer to the interests or convenience of other practitioners of Dental Surgery, I place my views on paper. This seems advisable also, as my opinions are radically opposed to what appears to be the settled purpose of the Census office. For the courtesy of your communication does not make it suggest any change of purpose since you first wrote to me, but rather a wish to learn the best means to accomplish it.

In regard to what is now called for in the schedule left with dentists, it is both improper to ask and impracticable to answer; and if the results obtained are embodied in the Census Report, it will excite ridicule and indignation, whereas by giving this up the Census officers will be put to less trouble, much money be saved to the country and the census report be more legitimate. This schedule and your letters to me demonstrate to my mind that the difficulty in the way of the Census officers is that they do not comprehend the specialty of Dental Surgery. When its nature is shown to them, they will, I think, see that it is unwise and also illegal to demand statements from dental practitioners, in respect to their professional doings.

You say to me: "The object of the inquiries submitted to you is to ascertain the amount of industry expended on the "mechanical" branch of Dentistry, and the form of schedule has been approved by the Census office." You then intimate that surgical operations may be excepted.

The word Surgery derived from Chirurgery denotes handwork in connection with the art of healing, and this whether manual or instrumental, or both conjointly.

Orthopedy (*Medical*) is the art of curing or remedying deformities in the human body.

Prothesis (*Surgical*) signifies the addition of some artificial part to the human body.

Marked examples in both of these classes are shown in the Report, edited by Professor Francis A. Walker, when Chief of the Bureau of Awards of the International Exhibition, 1876, and now Chief of the Census Office, Department of the Interior, Washington, D. C.

1st. A long description of the exhibits in the Dental Museum of the Noel Winderling Bros. of Milan, Italy; and on page 24 as now shown: "Then followed twenty-two specimens of *prothesis* and *orthopedy*, embracing pivot-teeth, dentures, complete and incomplete, with and without spiral springs, the various methods of retention by atmospheric pressure and by clasps, and a series of orthopedic apparatus for the correction of congenital malformations of the palate, and loss by scrofulous and syphilitic necrosis."

2nd. The report on "Hard rubber appliances for fractured jaws," opens as follows: "Mr. Thomas B. Gunning, of New York, exhibited a series of splints and appliances for the treatment of simple and compound fractures of the jaw."

Nearly four pages are given to their explanation, which closes thus:

"In connection with the splints, was a series of casts illustrating the double compound fracture of the jaw of the late Hon. William H. Seward, showing the jaw broken on both sides between the bicuspid teeth. Also a double cast of the upper and lower jaws as held by the splint for 68 days.

As no teeth were left in the upper jaw, the wings and cap were used as shown in Fig. 3.

The result was thoroughly satisfactory."

3rd. On page 28 "Hard rubber appliance for Congenital Cleft palate," the opening says: "This contrivance is a very marked improvement over all previous appliances for rectification of this distressing malformation." It was first applied by Dr. Gunning in 1864."

"These plates avoid the objectionable featuress of earlier appliances and to some extent enable the wearer to utilize the action of the muscles of the cleft velum."

These extracts are from the "General Report," certified as follows:

"International Exhibition,"
Philadelphia, 1876.

Prof. Francis A. Walker,
*Chief of Bureau of Awards.*

*Sir.*—I have the honor herewith to transmit the report of the Judges of Group XXIV, on Medicine, Surgery and Prothesis.

Respectfully Yours,
J. H. Thompson,
*Secretary Group XXIV.*

Of these Judges, one was Surgeon General, German Army, one from Austria, one from New Orleans, and the Secretary lived in Washington, D. C. No one of them was a dentist; they were all in Medicine and Surgery, and their testimony shows that these exhibits were surgical.

These appliances embrace or represent in principle all which dentists make or use in practice.

In support of my right and ability to state this, I submit that in 1866 I was appointed to decide upon the instruments and appliances for which space was asked in the Paris Universal Exhibition of 1867, as shown by the following extract from the Official Circular of the Department of State, Washington D. C.

"Class II. Medical Instruments.

Dr. J. K. Barnes, Surgeon General of the United States; Dr. Frank H. Hamilton, Professor of Surgery, Dr. Wm. H. Van Buren, Professor of Surgery; Dr. Ernest Krakowizer, Professor of Surgery; Dr. John M. Carnochan, Professor of Surgery; Dr. Thomas B. Gunning, Surgeon."

I have also been a practitioner of dental surgery in all its branches for over forty years; as shown in the Contemporary Biography of New York.

The agent of the Census Office, who called upon me, when saying I might except surgical operations, mentioned the extraction of teeth. Now as the extraction of teeth is surgical: inserting artificial ones to be used by the patient is not only more useful, but also surgical, according to the import of the surgical terms, hereinbefore presented.

Prothesis includes all forms of artificial teeth, with or without plates, and in every method of insertion, together with all constructions worn within the mouth, or any artificial addition to the human body.

Orthopedy includes appliances for congenital and acquired cleft palate, and for all malformations of the parts within and around the mouth, and for irregularity in the teeth &c.

To apply splints within the mouth in fracture or resections of the jaws, or for any other purpose, with or without external appliances, or these latter also, is surgery.

The appliances made in the operating rooms and laboratories of dentists which the Census Officers class as mechanical are made only after examination of the patient, or of the mouth, or parts for which the appliance is needed. In some cases teeth or other parts require removal or some additions; such as fillings, alterations in form, extraction, the removal of diseased bone, &c., and the healing of the parts.

In most cases the appliance cannot be made except by the aid of casts or impressions; these are taken off, or from each *particular patient or person*, for whom the appliance is necessary, and without whose co-operation the work cannot begin, and when the appliance is finished it is useless and of no value; except to or for that *particular patient.* These appliances are thus surgical; they are the results of diagnosis, prognosis and handwork combined to produce a useful result in, or appliance to, or for the human frame; this even when no medicine is given or prescribed, is surgery.

In filling teeth the result of the dentist's labor, is the addition of artificial parts to defective organs of the human body; this addition to each tooth of a patient being when completed virtually a part of that *particular person.* Filling teeth is therefore a surgical operation.

I have submitted only those exhibits, entered by dentists, which were remarked upon at length in the Official Report of the International Exhibition of 1876, edited by Professor Francis A. Walker, who is now Chief of the Census Office. One of these three exhibits was from Italy, two, my own, from New York. These exhibits as before stated included all the principles involved in the appliances used, and in those made by dentists for their patients, each appliance being made for and used only by the person for whom it is constructed.

The Report also remarked upon exhibits of a different class, viz.; of large and varied assortments of artificial teeth and of those which included all the materials and the manufactured articles made for and supplied to dental practitioners, who select what ever they require to be used in the different operations of their specialty.

These two distinctly different classes of exhibits, illustrate precisely the point at which the Census Office should stop in its demand for reports.

Those who make or sell whatever is, or may be, required by, and be useful for *many different persons* whether dentists or not; may perhaps be called upon to report.

Dentists and other specialists of Medical and Surgical art, who stand in delicate and confidential relations to their patients, and do for each one that which the case requires, should not be asked to report in respect to their professional doings; otherwise dental practitioners might be called upon to give information in regard to their personal treatment of any one of their patients

I have presented enough to show that the Dental Surgeon cannot properly be subjected to investigation and annoyance in respect to his professional work any more than the surgeon in other specialties of medical art: and the agent from the Census Office said that no schedule was left with physicians and surgeons; that they, lawyers and professional men were not asked to report; and your letter intimates that for surgical dentistry no statement is required.

I therefore protest against the novel and unjustifiable demand now made, for reports from Dental Practitioners.

I remain Sir, Your obedient servant,
THOMAS BRIAN GUNNING, D. D. S.

---

DEPARTMENT OF THE INTERIOR—CENSUS OFFICE.

*Office of Chief Special Agent,*
21 Courtland Street,
NEW YORK, DEC. 28th, 1880.

DR. T. B. GUNNING,
34 East 21st Street,
CITY.

*My Dear Sir.*—I have to thank you for your very courteous and interesting communication under date of the 24th., this morning at hand.

Its reception was most opportune, inasmuch as I was at the moment, closing an official letter to the Superintendent of the Census, discussing the same subject to which your letter relates, and which it so ably treats.

I have therefore transmitted it to him with my own, and await his definite reply, when I trust any difference of views which may exist between any of the members of your profession and the Census Office, will vanish.

Very Truly Yours, &c.,
CHARLES E. HILL,
*Chief Special Agent.*

---

DEPARTMENT OF THE INTERIOR—CENSUS OFFICE.

*Office of Chief Special Agent.*
21 Courtland Street,
NEW YORK, JAN. 4TH, 1881.

DR. T. B. GUNNING, D. D. S.
34 East 21st. Street,
NEW YORK.

*Dear Sir.*—Referring to my letter of the 28th. ultimo, I have now to say that the superintendent of the Census, upon the representation made to him by this office of the general reluctance of the Profession to give the information contemplated by the schedule, and upon consideration of the paper by yourself submitted to him, advises me (under date of yesterday,) that he has determined to omit from the tabulations of this office "Dentistry in all its branches."

All the schedules now in this office will be endorsed to this effect, and respectfully returned to their respective makers.

I am sir, Very Truly Yours,

Charles E. Hill,

*Chief Special Agent.*

